# Histamine-induced itch and its relationship with pain

**DOI:** 10.1186/1744-8069-4-29

**Published:** 2008-07-31

**Authors:** Won-Sik Shim, Uhtaek Oh

**Affiliations:** 1 National Research Laboratory of Transporters Targeted Drug Design, Research Institute of Pharmaceutical Sciences, College of Pharmacy, Seoul National University, Seoul, 151-742, Korea; 2 Sensory Research Center, College of Pharmacy, Seoul National University, Seoul, 151-742, Korea

## Abstract

Itch is one of the major complications of skin diseases. Although there are various substances that induce itch or pruritus, it is evident that histamine is the best known endogenous agent that evokes itch. Even though histamine-induced itch has been studied for some time, the underlying mechanism of itch is just beginning to emerge. Although various downstream signaling pathways of histamine receptors have been revealed, more studies are required to determine the cause of histamine-induced itch. It appears that itch and pain involve different neuronal pathways. Pain generally inhibits itch, which indicates an inter-communication between the two. Complex interactions between itch and pain may be expected based on reports on disease states and opioids. In this review, we discuss the molecular mechanism and the pharmacological aspects of histamine-induced itch. Especially, the underlying mechanism of TRPV1 (an anti-pruritus target) has been determined to some extent.

## Introduction

Itch is a sensation felt on skin, which causes the desire to scratch. Although itch might constitute an alert system against certain stimuli like mosquito bites, it can become stressful and exhausting when excessive. Indeed, patients with severe pruritus often find it difficult to lead a normal life due to itch-associated psychological disturbances, such as, depression or sleep deprivation [1,2]. Atopic dermatitis patients suffer from severe itch, and this disease is inadequately addressed by currently available medications. Therefore, an understanding of the mechanism of itch is essential in order to treat severe symptoms.

Although numerous substances are known to cause pruritus, such as, substance P, cytokines, proteases and so forth (for a detailed review on pruritogenic agents, see [3,4]), histamine is best known to evoke experimental itch when applied to the skin [5-9]. Recent itch-related studies have focused on non-histaminergic itch symptoms, but it is also of considerable importance that we understand the manner in which histamine induces itch. In this regard, it is worth mentioning that antihistamines are among the most widely-used drugs in the United States [10]. Therefore, in this review, we will focus mainly on experimental findings concerning histamine-induced itch.

### Histamine and itch

Histamine is released from mast cells when tissues are inflamed or stimulated by allergens [11,12], and once released, histamine induces itch is triggered by the excitation of a subset of unmyelinated C-fibers [13]. Histamine receptors are known to mediate histamine-induced responses, and are members of the G-protein-coupled receptors. Four subtypes of histamine receptors have been identified to date, and histamine receptor subtype I (H1R) has been studied most extensively in the context of histamine-induced itch. In fact, H1R blockers (antihistamines) are widely used to manage and alleviate itch symptoms [14]. However, the itch-reducing efficacies of these classical H1R antihistamines are debatable because some believe that the effect is attributable to sedation rather than to H1R antagonism [15]. It appears that H1R antagonism does, at least to some extent, attenuate histamine-induced itch, because non-sedative second generation H1R antihistamines are beneficial for the management of itch symptoms [16]. However, in contrast to the proven relation between H1R and itch induction, the involvement of histamine receptor subtype II (H2R) is less convincing. It is generally believed that H2R is at best, only marginally involved in histamine-induced itch process [17,18]. For instance, dimaprit (a H2R agonist) failed to cause scratching, and cimetidine (a H2R antagonist) failed to suppress histamine-induced itch in BalbC mice [19]. On the other hand, it is intriguing that histamine receptor subtype III (H3R) "*antagonists*" aggravate itch symptoms, which appears to contradict the aforementioned histamine-induced itch pathway [20]. For example, the blockade of H3R by H3R-specific antagonists (thioperamide or AQ0145) was found to significantly increase the incidence of scratching behavior in mice [21]. Furthermore, intradermal injections of iodophenpropit or clobenpropit (also H3R antagonists) caused significant increases in scratching behavior in both mast cell-deficient and wild-type mice [22]. Currently, it appears that the itch elicited by H3 antagonism is mediated by substance P, another itch-inducing agent [23]. However, it could also be mediated by mixed responses from H3R and histamine receptor subtype IV (H4R), since clobenpropit (a H3R antagonist) is also an agonist of H4R [24]. H4R agonists cause scratching responses in mice, and are attenuated by pretreating animals with a selective H4R antagonist, like JNJ7777120 [25]. It is also noteworthy that scratching behaviors are almost completely abolished when H1R/H4R antagonists or H1 antagonist are co-administered to H4R-knockout mice, which suggests that H1R and H4R are key components of the itch response [25].

Summarizing, it appears that activated H1R and H4R are involved in the induction of itch, whereas H3R acts in the reverse manner. On the other hand, it appears that H2R has a minor role at most.

### The histamine signaling pathway in sensory neurons

H1R is coupled with Gα_q _proteins, and this interaction activates phospholipase C (PLC) [26]. In line with this, it has been reported that histamine elevates calcium levels in rat cultured sensory neurons, and that this elevation is blocked by U73122 (a PLC inhibitor) [27]. Moreover, it was recently found that PLCβ3 (and not the other PLC isotypes) specifically mediates histamine-induced calcium responses via H1R in cultured sensory neurons [28]. On the other hand, stimulation of phospholipase A_2 _(PLA_2_) by H1R was found to mediate histamine-induced sensory neuron excitation [29,30]. Furthermore, Shim and colleagues showed that histamine induces itch by activating PLA_2_, lipoxygenase, and the TRPV1 signaling pathway [30]. Histamine induces inward currents that are blocked by antagonists of TRPV1, a nonselective cation channel stimulated by capsaicin [30,31]. They also demonstrated that TRPV1 activation in sensory neurons by histamine is mediated by the synthesis of 12-HPETE – a metabolite of 12-lipoxygenase and an endogenous TRPV1 activator [30,32]. Moreover, histamine-induced scratching is greatly reduced when inhibitors or blockers of H1R, PLA_2_, 12-lipoxygenase, or TRPV1 have been pre-treated. More importantly, histamine-induced scratching is significantly lower in TRPV1-deficient mice [30]. In line with this, the direct intradermal injection of 12-HPETE (an endogenous TRPV1 activator) was found to evoke scratching behaviors in mice [33]. However, this 12-HPETE/TRPV1 linkage is somewhat controversial, since 12-HPETE-induced scratching in mice is not blocked by the TRPV1 antagonist capsazepine [34]. However, 12-HPETE-induced scratching is reduced by topical application of capsaicin [33], which is also known to cause desensitization of TRPV1. More studies are required to determine whether 12-HPETE/TRPV1 activation mechanisms participate in H1R-related itch pathways.

It is well-known that H2R is involved in gastric acid secretion, wherein the coupling of H2R and Gα_s _leads to cAMP production [26]. However, as mentioned above, the role of H2R in itch appears minor. H3R, on the other hand, is linked to Gα_i/o _and the activation of H3R mainly inhibits cAMP formation [35], but various down-stream signals are also generated, such as, the activations of mitogen-activated protein kinase (MAPK), PLA_2_, and others (For a detailed review, see [36]). However, it should be noted that H3R is predominantly expressed in the central nervous system, and was first identified in brain [37]. Although its existence in perivascular nerve terminals has been suggested [38], it appears that H3R is not present in the peripheral nervous system – at least in mice [23]. Therefore, the role of H3R in the mediation of histamine-induced itch seems minor. However, interestingly, H3R is regarded as a novel target for the treatment of obesity and cognitive disorders [39,40].

The importance of H4R in terms of itch is becoming more evident [41,42]. It is well known that the activation of H4R increases intracellular Ca^2+ ^levels; possibly in a phospholipase C-dependent manner in mast cells [43]. Moreover, the existence of H4R in sensory neurons is suggested by the observation that an intradermally administered H4R-specific agonist elicited scratching in mice, which were completely inhibited by pretreatment of H4R antagonist, JNJ7777120 [25]. Thus, it seems likely that the activation of H4R results in the excitation of itch-mediating histamine-sensitive afferents by increasing intracellular Ca^2+ ^levels, as mentioned above for the H1R pathway.

### Neurophysiology of itch fibers

Most C fibers are polymodal nociceptors that respond to noxious mechanical and heat stimuli (CMH units). These CMH units are mainly related to nociception, but are largely insensitive to or only weakly activated by histamine [44]. In addition, histamine-sensitive C-fibers do not respond to mechanical stimulation, which indicates that these itch-mediating fibers differ from polymodal C-fibers [45]. Indeed, these itch-mediating C fibers only comprise about five percent of afferent C-fibers in human cutaneous nerves [5]. Histamine activates a subset of C-fibers that innervate the superficial layer of skin and transmit electrical signals to the superficial layer of the dorsal horn of the spinal cord [5]. These signals then ascend to the thalamus through contralateral spinothalamic tracts and are eventually conducted to the somatosensory and cingulate cortex [46]. Interestingly, it was found that gastrin-releasing peptide plays a key role in mediating itch sensation, rather than pain, by interacting with gastrin-releasing peptide receptor (GRPR) at the spinal level [47]. Furthermore, the induction of scratching behavior in response to pruritogenic stimuli was significantly diminished in GRPR knockout mice, but pain-related behavioral responses to noxious stimuli were normal [47]. In addition, direct spinal injection of a GRPR antagonist considerably inhibited scratching behaviors [47]. Again, these results provide support for the presence of a distinct itch-mediating pathway. On the other hand, the itch elicited by cowhage (a non-histaminergic pruritogen) appears to be mediated through other distinct primary afferents [48] and through cowhage-specific non-histaminergic spinothalamic tracts [49]. Therefore, it seems that different types of itch-mediating neurons coexist in the periphery. Recently, the active compound in cowhage was identified as a novel cysteine protease "mucunain", which is a ligand for protease-activated receptors-2 and 4 [50]. Nakano *et al *recently similarly concluded that different types of dorsal horn neurons are associated with histamine-induced and protease-activated receptor-2-mediated itch [51]. Thus, it seems that there exist dedicated itch-mediating neuronal pathways. Moreover, the recent identification of the co-existence of histamine-sensitive and insensitive (or protease-related) pathways may provide some insight into the mechanism of itch.

### Painful stimuli inhibits itch sensation

We all share the experience that scratching relieves itching. Furthermore, itch is also relieved when noxious heat is administered [52]. In other words, itch can be suppressed by painful mechanical and thermal stimuli. As stated above, itch- and pain-inducing stimuli activate distinct populations of sensory fibers, and thus, it is likely that painful stimuli modulate itch sensations centrally and not at the peripheral level. Histamine was found not to induce itch when a noxious thermal stimulus was administered within 10 cm, or to reduce itch severity if a noxious stimulus was administered more than 10 cm away [53]. Noxious cold also reduces pruritus when administered to fingertips contralateral to a pruritic stimulus [54]. Moreover, various other painful stimuli, such as, noxious heat or scratching, are known to inhibit histamine-induced itch via central mechanisms [55-57].

In fact, capsaicin, the active ingredient of hot pepper, is used as an anti-pruritic agent [58]. Capsaicin activates the nonselective cation channel TRPV1, which is regarded to induce nociception [31]. Thus, it is assumed that the anti-pruritic effect of capsaicin is attributable to its algesic effect. However, it should be noted that TRPV1 is expressed in some histamine-sensitive itch-mediating fibers as well as nociceptive C-fibers [30], which raises the question; What types of processes are involved in the sensation experienced when capsaicin is applied to the skin? In most cases, pain is likely to be the predominant perception, since approximately 80% of the primary afferent C-fibers are polymodal nociceptors [59-61]. In fact, the clinical limitation of topical capsaicin administered as an anti-pruritic agent is that it produces unbearable, burning pain [58]. Furthermore, even if capsaicin excites TRPV1 in itch fibers, the itch sensation may not dominate since itch-mediating fibers comprise only small proportion of C fibers [5]. Thus, although capsaicin may activate both pain and itch through TRPV1 receptors in their respective neurons, it is highly likely that capsaicin preferentially activates nociceptive fibers.

However, it should also be noted that repeated and prolonged applications of topical capsaicin are required to effectively reduce itch [62,63]. This method of application is believed to fully desensitize and deplete neuropeptides, such as, substance P in sensory afferents, and to thus delay the interconnection between skin and sensory neurons [58]. Indeed, it has been shown that repetitive application of topical capsaicin prevents histamine-induced itch under experimental conditions [64]. In this regard, it can also be considered that the anti-pruritic effect of capsaicin may stem from peripheral desensitization of sensory neurons and central mechanisms.

Itch can also be suppressed by cold stimuli [65-67], and in particular the anti-pruritic effect of menthol is interesting [65], because menthol activates cold receptor TRPM8 [68,69]. However, although TRPM8 is a wonderful molecular target, the mechanism whereby cold and menthol mitigates itch has yet to be determined. TRPA1, which is activated by noxious cold (<17°C), is also a viable target [70]. but no concrete relationship between itch and TRPA1 has been established. On the other hand, warming appears to aggravate itch. Indeed, histamine-induced response is potentiated by warming [67], but no clear explanation has been offered as to how these thermal stimuli interact with itch at the molecular level. It is noteworthy that some TRP channels, like TRPV3 [71,72] respond to warming, which suggests that they participate in itch induction. However, no studies to date have focused specifically on this topic.

Unfortunately, the relationships between itch and exogenous stimuli in disease states appear anything but straightforward. For instance, the itch-inhibitory effects of repetitive scratching and noxious heat are ineffective in patients with atopic dermatitis [73]. Similarly, the inhibitory effect of topical capsaicin on histamine-induced itch was found to be ineffective in atopic dermatitis patients, but effective in healthy controls [64], indicating that other factors are involved in disease states. Moreover, in contrast to our general understanding that cooling alleviates itch, short-term low-intensity cooling increases the intensity of histamine-induced itch above the scratch threshold in man [74,75].

Interestingly, as a corollary to the suppression of itch by painful stimuli, the reduction of pain by opioids may induce itch [76]. Patients spinally administered μ-opioid agonists frequently experience itch [77,78], whereas μ-opioid antagonists often suppress experimentally-induced itch [8,79]. However, not all opioids evoke itch, for example, nalbuphine (a κ-opioid agonist) has been shown to reduce μ-opioid-induced pruritus [80], and κ-opioid antagonists enhance itch, which contrasts the effects of μ-opioids [81]. Currently, it is unclear why different opioids have different effects on itch. Nevertheless, it seems evident that itch can be enhanced when pain is suppressed, and suppressed when pain is enhanced, which demonstrates the existence of an intimate physiologic interaction between underlying causes of itch and pain sensations.

## Conclusion

Itch is probably viewed as trivial malady by most, but to many patients itch is a distressing condition. Although it has been revealed by many researchers that there is a histamine-independent itch, this should not detract from the fact that histamine is deeply involved in various itch sensations. Recent advances in molecular biology have helped reveal the key molecular players involved, but a considerable amount of effort will be required to determine how histamine-induced itch is mediated and can be inhibited. In our opinion, a thorough understanding of the pruritogenic actions of *histamine *is required if we are to resolved itch symptoms at the clinical level.

## Competing interests

The authors declare that they have no competing interests.

## Authors' contributions

WS drafted the manuscript and UO revised the manuscript.

## References

[B1] Hashiro M, Okumura M (1997). Anxiety, depression and psychosomatic symptoms in patients with atopic dermatitis: comparison with normal controls and among groups of different degrees of severity. J Dermatol Sci.

[B2] Sheehan-Dare RA, Henderson MJ, Cotterill JA (1990). Anxiety and depression in patients with chronic urticaria and generalized pruritus. Br J Dermatol.

[B3] Paus R, Schmelz M, Biro T, Steinhoff M (2006). Frontiers in pruritus research: scratching the brain for more effective itch therapy. J Clin Invest.

[B4] Ikoma A, Steinhoff M, Stander S, Yosipovitch G, Schmelz M (2006). The neurobiology of itch. Nat Rev Neurosci.

[B5] Schmelz M, Schmidt R, Bickel A, Handwerker HO, Torebjork HE (1997). Specific C-receptors for itch in human skin. J Neurosci.

[B6] Schmelz M, Schmidt R, Weidner C, Hilliges M, Torebjork HE, Handwerker HO (2003). Chemical response pattern of different classes of C-nociceptors to pruritogens and algogens. J Neurophysiol.

[B7] Magerl W, Westerman RA, Mohner B, Handwerker HO (1990). Properties of transdermal histamine iontophoresis: differential effects of season, gender, and body region. J Invest Dermatol.

[B8] Heyer G, Dotzer M, Diepgen TL, Handwerker HO (1997). Opiate and H1 antagonist effects on histamine induced pruritus and alloknesis. Pain.

[B9] Ikoma A, Rukwied R, Stander S, Steinhoff M, Miyachi Y, Schmelz M (2003). Neuronal sensitization for histamine-induced itch in lesional skin of patients with atopic dermatitis. Arch Dermatol.

[B10] Kaufman DW, Kelly JP, Rosenberg L, Anderson TE, Mitchell AA (2002). Recent patterns of medication use in the ambulatory adult population of the United States: the Slone survey. JAMA.

[B11] Benditt EP, Bader S, Lam KB (1955). Studies of the mechanism of acute vascular reactions to injury. I. The relationship of mast cells and histamine to the production of edema by ovomucoid in rats. AMA Arch Pathol.

[B12] Rowley DA, Benditt EP (1956). 5-Hydroxytryptamine and histamine as mediators of the vascular injury produced by agents which damage mast cells in rats. J Exp Med.

[B13] Tani E, Shiosaka S, Sato M, Ishikawa T, Tohyama M (1990). Histamine acts directly on calcitonin gene-related peptide- and substance P-containing trigeminal ganglion neurons as assessed by calcium influx and immunocytochemistry. Neurosci Lett.

[B14] Simons FE, Simons KJ, Becker AB, Haydey RP (1984). Pharmacokinetics and antipruritic effects of hydroxyzine in children with atopic dermatitis. J Pediatr.

[B15] Krause L, Shuster S (1983). Mode of action of H1 antihistamines in itch. Br J Dermatol.

[B16] Katagiri K, Arakawa S, Hatano Y, Fujiwara S (2006). Fexofenadine, an H1-receptor antagonist, partially but rapidly inhibits the itch of contact dermatitis induced by diphenylcyclopropenone in patients with alopecia areata. J Dermatol.

[B17] Hagermark O, Strandberg K, Gronneberg R (1979). Effects of histamine receptor antagonists on histamine-induced responses in human skin. Acta Derm Venereol.

[B18] Davies MG, Greaves MW (1980). Sensory responses of human skin to synthetic histamine analogues and histamine. Br J Clin Pharmacol.

[B19] Bell JK, McQueen DS, Rees JL (2004). Involvement of histamine H4 and H1 receptors in scratching induced by histamine receptor agonists in Balb C mice. Br J Pharmacol.

[B20] Summey BT, Yosipovitch G (2005). Pharmacologic advances in the systemic treatment of itch. Dermatol Ther.

[B21] Sugimoto Y, Iba Y, Nakamura Y, Kayasuga R, Kamei C (2004). Pruritus-associated response mediated by cutaneous histamine H3 receptors. Clin Exp Allergy.

[B22] Hossen MA, Sugimoto Y, Kayasuga R, Kamei C (2003). Involvement of histamine H3 receptors in scratching behaviour in mast cell-deficient mice. Br J Dermatol.

[B23] Hossen MA, Inoue T, Shinmei Y, Fujii Y, Watanabe T, Kamei C (2006). Role of substance P on histamine H(3) antagonist-induced scratching behavior in mice. J Pharmacol Sci.

[B24] Oda T, Morikawa N, Saito Y, Masuho Y, Matsumoto S (2000). Molecular cloning and characterization of a novel type of histamine receptor preferentially expressed in leukocytes. J Biol Chem.

[B25] Dunford PJ, Williams KN, Desai PJ, Karlsson L, McQueen D, Thurmond RL (2007). Histamine H4 receptor antagonists are superior to traditional antihistamines in the attenuation of experimental pruritus. J Allergy Clin Immunol.

[B26] Bakker RA, Timmerman H, Leurs R (2002). Histamine receptors: specific ligands, receptor biochemistry, and signal transduction. Clin Allergy Immunol.

[B27] Nicolson TA, Bevan S, Richards CD (2002). Characterisation of the calcium responses to histamine in capsaicin-sensitive and capsaicin-insensitive sensory neurones. 0306-4522.

[B28] Han SK, Mancino V, Simon MI (2006). Phospholipase Cbeta 3 mediates the scratching response activated by the histamine H1 receptor on C-fiber nociceptive neurons. 0896-6273.

[B29] Kim BM, Lee SH, Shim WS, Oh U (2004). Histamine-induced Ca(2+) influx via the PLA(2)/lipoxygenase/TRPV1 pathway in rat sensory neurons. Neurosci Lett.

[B30] Shim WS, Tak MH, Lee MH, Kim M, Kim M, Koo JY, Lee CH, Kim M, Oh U (2007). TRPV1 mediates histamine-induced itching via the activation of phospholipase A2 and 12-lipoxygenase. J Neurosci.

[B31] Caterina MJ, Schumacher MA, Tominaga M, Rosen TA, Levine JD, Julius D (1997). The capsaicin receptor: a heat-activated ion channel in the pain pathway. 0028-0836.

[B32] Hwang SW, Cho H, Kwak J, Lee SY, Kang CJ, Jung J, Cho S, Min KH, Suh YG, Kim D, Oh U (2000). Direct activation of capsaicin receptors by products of lipoxygenases: endogenous capsaicin-like substances. Proc Natl Acad Sci USA.

[B33] Kim DK, Kim HJ, Sung KS, Kim H, Cho SA, Kim KM, Lee CH, Kim JJ (2007). 12(S)-HPETE induces itch-associated scratchings in mice. Eur J Pharmacol.

[B34] Kim DK, Kim HJ, Kim H, Koh JY, Kim KM, Noh MS, Kim JJ, Lee CH (2008). Involvement of serotonin receptors 5-HT1 and 5-HT2 in 12(S)-HPETE-induced scratching in mice. Eur J Pharmacol.

[B35] Leurs R, Bakker RA, Timmerman H, de Esch IJ (2005). The histamine H3 receptor: from gene cloning to H3 receptor drugs. Nat Rev Drug Discov.

[B36] Bongers G, Bakker RA, Leurs R (2007). Molecular aspects of the histamine H3 receptor. Biochem Pharmacol.

[B37] Arrang JM, Garbarg M, Schwartz JC (1983). Auto-inhibition of brain histamine release mediated by a novel class (H3) of histamine receptor. 0028-0836.

[B38] Ishikawa S, Sperelakis N (1987). A novel class (H3) of histamine receptors on perivascular nerve terminals. 0028-0836.

[B39] Esbenshade TA, Fox GB, Cowart MD (2006). Histamine H3 receptor antagonists: preclinical promise for treating obesity and cognitive disorders. Mol Interv.

[B40] Esbenshade TA, Browman KE, Bitner RS, Strakhova M, Cowart MD, Brioni JD (2008). The histamine H(3) receptor: an attractive target for the treatment of cognitive disorders. Br J Pharmacol.

[B41] Huang JF, Thurmond RL (2008). The new biology of histamine receptors. Curr Allergy Asthma Rep.

[B42] Thurmond RL, Gelfand EW, Dunford PJ (2008). The role of histamine H1 and H4 receptors in allergic inflammation: the search for new antihistamines. Nat Rev Drug Discov.

[B43] Hofstra CL, Desai PJ, Thurmond RL, Fung-Leung WP (2003). Histamine H4 receptor mediates chemotaxis and calcium mobilization of mast cells. J Pharmacol Exp Ther.

[B44] Handwerker HO, Forster C, Kirchhoff C (1991). Discharge patterns of human C-fibers induced by itching and burning stimuli. J Neurophysiol.

[B45] Schmelz M, Schmid R, Handwerker HO, Torebjörk HE (2000). Encoding of burning pain from capsaicin-treated human skin in two categories of unmyelinated nerve fibres. 0006-8950.

[B46] Andrew D, Craig AD (2001). Spinothalamic lamina I neurons selectively sensitive to histamine: a central neural pathway for itch. Nat Neurosci.

[B47] Sun YG, Chen ZF (2007). A gastrin-releasing peptide receptor mediates the itch sensation in the spinal cord. 0028-0836.

[B48] Johanek LM, Meyer RA, Hartke T, Hobelmann JG, Maine DN, LaMotte RH, Ringkamp M (2007). Psychophysical and physiological evidence for parallel afferent pathways mediating the sensation of itch. J Neurosci.

[B49] Davidson S, Zhang X, Yoon CH, Khasabov SG, Simone DA, Giesler GJ (2007). The itch-producing agents histamine and cowhage activate separate populations of primate spinothalamic tract neurons. J Neurosci.

[B50] Reddy VB, Iuga AO, Shimada SG, LaMotte RH, Lerner EA (2008). Cowhage-evoked itch is mediated by a novel cysteine protease: a ligand of protease-activated receptors. J Neurosci.

[B51] Nakano T, Andoh T, Lee JB, Kuraishi Y (2008). Different dorsal horn neurons responding to histamine and allergic itch stimuli. 0959-4965.

[B52] Schmelz M (2002). Itch – mediators and mechanisms. J Dermatol Sci.

[B53] Bickford RG (1937). Experiments relating to the itch sensation, its peripheral mechanism, and central pathways. Clin Sci.

[B54] Murray FS, Weaver MM (1975). Effects of ipsilateral and contralateral counterirritation on experimentally produced itch in human beings. J Comp Physiol Psychol.

[B55] Fruhstorfer H, Hermanns M, Latzke L (1986). The effects of thermal stimulation on clinical and experimental itch. Pain.

[B56] Ward L, Wright E, McMahon SB (1996). A comparison of the effects of noxious and innocuous counterstimuli on experimentally induced itch and pain. Pain.

[B57] Yosipovitch G, Duque MI, Fast K, Dawn AG, Coghill RC (2007). Scratching and noxious heat stimuli inhibit itch in humans: a psychophysical study. Br J Dermatol.

[B58] Biro T, Toth BI, Marincsak R, Dobrosi N, Geczy T, Paus R (2007). TRP channels as novel players in the pathogenesis and therapy of itch. Biochim Biophys Acta.

[B59] Schmelz M (2001). A neural pathway for itch. Nat Neurosci.

[B60] Schmidt R, Schmelz M, Forster C, Ringkamp M, Torebjork E, Handwerker H (1995). Novel classes of responsive and unresponsive C nociceptors in human skin. J Neurosci.

[B61] Schmidt R, Schmelz M, Ringkamp M, Handwerker HO, Torebjork HE (1997). Innervation territories of mechanically activated C nociceptor units in human skin. J Neurophysiol.

[B62] Lysy J, Sistiery-Ittah M, Israelit Y, Shmueli A, Strauss-Liviatan N, Mindrul V, Keret D, Goldin E (2003). Topical capsaicin – a novel and effective treatment for idiopathic intractable pruritus ani: a randomised, placebo controlled, crossover study. 0017-5749.

[B63] Stander S, Luger T, Metze D (2001). Treatment of prurigo nodularis with topical capsaicin. J Am Acad Dermatol.

[B64] Weisshaar E, Heyer G, Forster C, Handwerker HO (1998). Effect of topical capsaicin on the cutaneous reactions and itching to histamine in atopic eczema compared to healthy skin. Arch Dermatol Res.

[B65] Bromm B, Scharein E, Darsow U, Ring J (1995). Effects of menthol and cold on histamine-induced itch and skin reactions in man. Neurosci Lett.

[B66] Carstens E, Jinks SL (1998). Skin cooling attenuates rat dorsal horn neuronal responses to intracutaneous histamine. 0959-4965.

[B67] Mizumura K, Koda H (1999). Potentiation and suppression of the histamine response by raising and lowering the temperature in canine visceral polymodal receptors in vitro. Neurosci Lett.

[B68] McKemy DD, Neuhausser WM, Julius D (2002). Identification of a cold receptor reveals a general role for TRP channels in thermosensation. 0028-0836.

[B69] Peier AM, Moqrich A, Hergarden AC, Reeve AJ, Andersson DA, Story GM, Earley TJ, Dragoni I, McIntyre P, Bevan S, Patapoutian A (2002). A TRP channel that senses cold stimuli and menthol. 0092-8674.

[B70] Story GM, Peier AM, Reeve AJ, Eid SR, Mosbacher J, Hricik TR, Earley TJ, Hergarden AC, Andersson DA, Hwang SW, McIntyre P, Jegla T, Bevan S, Patapoutian A (2003). ANKTM1, a TRP-like channel expressed in nociceptive neurons, is activated by cold temperatures. 0092-8674.

[B71] Smith GD, Gunthorpe MJ, Kelsell RE, Hayes PD, Reilly P, Facer P, Wright JE, Jerman JC, Walhin JP, Ooi L, Egerton J, Charles KJ, Smart D, Randall AD, Anand P, Davis JB (2002). TRPV3 is a temperature-sensitive vanilloid receptor-like protein. 0028-0836.

[B72] Xu H, Ramsey IS, Kotecha SA, Moran MM, Chong JA, Lawson D, Ge P, Lilly J, Silos-Santiago I, Xie Y, DiStefano PS, Curtis R, Clapham DE (2002). TRPV3 is a calcium-permeable temperature-sensitive cation channel. 0028-0836.

[B73] Ishiuji Y, Coghill RC, Patel TS, Dawn A, Fountain J, Oshiro Y, Yosipovitch G (2008). Repetitive scratching and noxious heat do not inhibit histamine-induced itch in atopic dermatitis. Br J Dermatol.

[B74] Pfab F, Valet M, Sprenger T, Toelle TR, Athanasiadis GI, Behrendt H, Ring J, Darsow U (2006). Short-term alternating temperature enhances histamine-induced itch: a biphasic stimulus model. J Invest Dermatol.

[B75] Valet M, Pfab F, Sprenger T, Woller A, Zimmer C, Behrendt H, Ring J, Darsow U, Tolle TR (2008). Cerebral processing of histamine-induced itch using short-term alternating temperature modulation – an FMRI study. J Invest Dermatol.

[B76] Atanassoff PG, Brull SJ, Zhang J, Greenquist K, Silverman DG, Lamotte RH (1999). Enhancement of experimental pruritus and mechanically evoked dysesthesiae with local anesthesia. Somatosens Mot Res.

[B77] Chaney MA (1995). Side effects of intrathecal and epidural opioids. Can J Anaesth.

[B78] Kam PC, Tan KH (1996). Pruritus – itching for a cause and relief?. 0003-2409.

[B79] Ko MC, Song MS, Edwards T, Lee H, Naughton NN (2004). The role of central mu opioid receptors in opioid-induced itch in primates. J Pharmacol Exp Ther.

[B80] Kjellberg F, Tramer MR (2001). Pharmacological control of opioid-induced pruritus: a quantitative systematic review of randomized trials. Eur J Anaesthesiol.

[B81] Kamei J, Nagase H (2001). Norbinaltorphimine, a selective kappa-opioid receptor antagonist, induces an itch-associated response in mice. Eur J Pharmacol.

